# FBXO22 Suppresses Metastasis in Human Renal Cell Carcinoma via Inhibiting MMP-9-Mediated Migration and Invasion and VEGF-Mediated Angiogenesis

**DOI:** 10.7150/ijbs.69330

**Published:** 2022-01-01

**Authors:** Feng Guo, Jinjin Liu, Xiao Han, Xuping Zhang, Tian Lin, You Wang, Jin Bai, Junqing Han

**Affiliations:** 1 Cancer Center, Shandong Provincial Hospital affiliated to Shandong University, Jinan 250021, Shandong Province, China.; 2 Cancer Institute, Xuzhou Medical University, Xuzhou 221002, Jiangsu Province, China.; 3 Department of Radiation Oncology, Xuzhou Cancer Hospital, Xuzhou 221005, Jiangsu Province, China.; 4 Department of Obstetrics and Gynecology, Renji Hospital, School of Medicine, Shanghai Jiao Tong University, Shanghai 200127, China.; 5 Department of Experiment, Tumor Hospital Affiliated to Guangxi Medical University, Nanning 530021, Guangxi Province, China.

Following the publication of our article, the authors noted one error in Fig. 3A. One of the authors saved all the migration and invasion images in the same fold when he performing the experiments in June 18^th^, 2015. After couples of years, another author started to make figures, he selected the wrong images because of his carelessness. In the old Fig. 3A, all the 4 images were from invasion experiment. In the new Fig. 3A, we replaced the two wrong images in the migration group. The authors confirm that the mistake does not affect the results or conclusions of the study and apologize for any inconvenience caused by this mistake. The old and new figures are provided side-by-side.

## Figures and Tables

**Figure 3:**
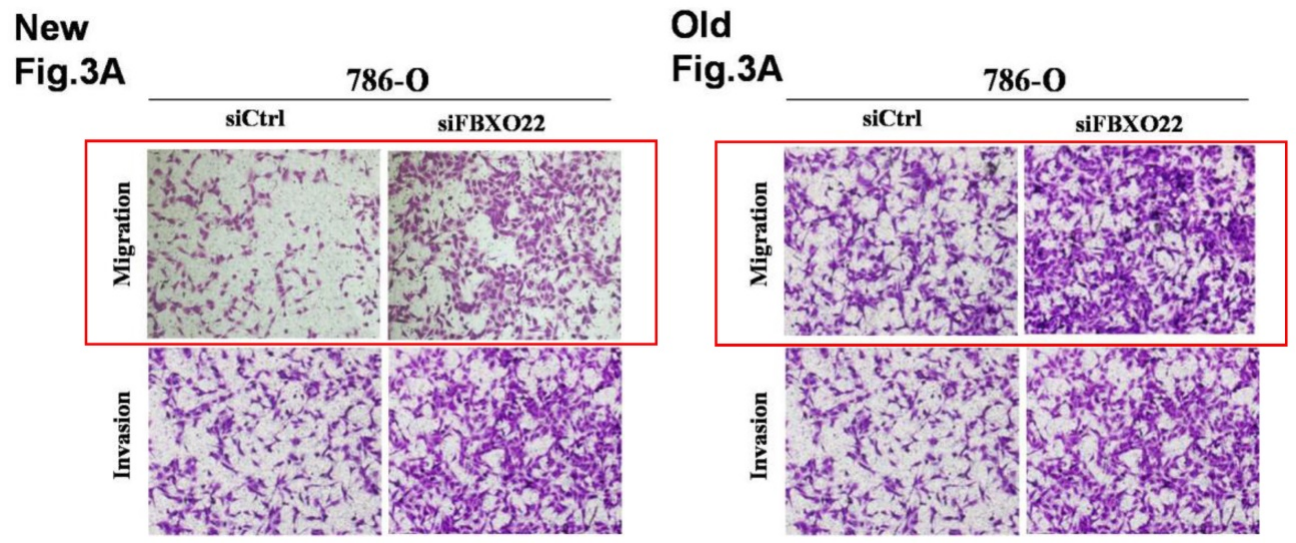
A. Correction.

